# Experimental Research and Numerical Simulation of Laser Welding of 303Cu/440C-Nb Stainless-Steel Thin-Walled Natural-Gas Injector for Vehicles

**DOI:** 10.3390/ma16052109

**Published:** 2023-03-05

**Authors:** Lisen Zhou, Dongya Li, Chonghai Xu, Zhaoxing Zheng, Yu Liu

**Affiliations:** 1 Faculty of Mechanical Engineering, Qilu University of Technology (Shandong Academy of Sciences), Jinan 250353, China; 2 School of Mechanical Engineering, Jiangnang University, Wuxi 214122, China; 3 Wuxi Longsheng Technology Co., Ltd., Wuxi 214028, China

**Keywords:** laser welding, austenitic/martensitic stainless-steel welding, numerical simulation, helium leakage test, laser post-heat treatment process

## Abstract

This paper presents the results of research on laser lap welding technology of heterogeneous materials and a laser post-heat treatment method to enhance welding performance. The purpose of this study is to reveal the welding principle of austenitic/martensitic dissimilar stainless-steel materials (3030Cu/440C-Nb) and to further obtain welded joints with good mechanical and sealing properties. A natural-gas injector valve is taken as the study case where its valve pipe (303Cu) and valve seat (440C-Nb) are welded. Experiments and numerical simulations were conducted where the welded joints’ temperature and stress fields, microstructure, element distribution, and microhardness were studied. The results showed that the residual equivalent stresses and uneven fusion zone tend to concentrate at the joint of two materials within the welded joint. The hardness of the 303Cu side (181.8 HV) is less than the 440C-Nb side (266 HV) in the center of the welded joint. The laser post-heat treatment can reduce the residual equivalent stress in the welded joint and improve the mechanical and sealing properties. The results of the press-off force test and the helium leakage test showed that the press-off force increased from 9640 N to 10,046 N and the helium leakage rate decreased from 3.34 × 10^−4^ to 3.96 × 10^−6^.

## 1. Introduction

In recent years, the proportion of natural gas in public transportation, freight logistics, and marine fuel has been increasing because of its high environmental protection, low economic cost, and abundant resource supply. As the core component of the engine, the natural-gas injector has a broad market demand under the background of continuous policy support and technology maturity [1]. The valve seat and pipe in the injector are to be lap-welded using welding technology. Compared to other welding technologies, laser welding has unique advantages in the application of injectors. Laser welding has a relatively strong heat penetration ability and a high weld depth–width ratio [2]. The input heat can be reduced to the minimum demand, and the thermal deformation is small [3]. The laser beam can be focused on a small area for small injectors, and the positioning is relatively accurate [4]. Laser welding has no concerns about electromagnetic pollution and will not affect the electromagnetic control performance of the injector [5]. Laser welding easily carries out high-speed welding automatically and has high welding efficiency. Furthermore, because it is not a contact welding process, the wear and deformation of machines and tools can be minimized [6,7,8].

Austenitic stainless-steel 303Cu is used as a material for the valve pipe in injectors due to its good corrosion resistance, durability, and ease of processing. Martensitic stainless steel 440C-Nb is used as the material of the valve seat because of its corrosion resistance and high strength. There are differences in the alloying elements, thermal conductivity, and coefficient of thermal expansion between austenitic and martensitic stainless steels [9]. These differences lead to the formation of hot cracks, carbon migration, brittle intermetallic compounds, and δ ferrite in the transition region of weld metal during welding [10]. It is also easy to form a soft alternating heat-affected zone (HAZ) on the martensitic stainless-steel side, which leads to problems related to element diffusion and residual stress [11,12]. Sharifitabar et al. [13] studied the resistance butt welding of austenitic stainless steel (AISI 304) to martensitic stainless steel (AISI 420), where an intermediate layer consisting of 80% ferrite and 20% martensite was formed at the welded joint interface. Casalino et al. [14] demonstrated good weldability of different austenitic and martensitic stainless steels under annealed conditions by welding austenitic stainless steel (AISI 304) and martensitic stainless steel (AISI 410) with a fiber laser TIG hybrid welding system. Dak et al. [15] studied heterogeneous laser welded joints of 304L austenitic stainless steel and P92 martensitic stainless steel, describing the weld metal microstructure, interface, and HAZ and observed a significant degree of inhomogeneity in the heterogeneous joints. Under the as-welded and post-weld heat treatment conditions, the welded joint shows a good combination of tensile and impact strength. Varbai et al. [16] studied dissimilar joints of LDX 2101 duplex stainless steel and 304 Austenitic stainless steel, evaluated the weld metal ferrite content and chemical composition according to the prediction diagrams, and obtained a good correlation with the experiments. Tuz et al. [17] studied the laser beam butt welding and post-weld heat treatment of a 17-4PH stainless-steel plate and revealed that the homogenization process took place in the welded joint area after heat treatment and affected the mechanical properties of the joint.

Many scholars have tried various welding processes to achieve the welding of different types of austenitic stainless steel and martensitic stainless steel [18,19,20]. However, the welding process for 303Cu and 440C-Nb has yet to be studied. Good mechanical and sealing properties after welding are crucial for laser welding valve pipes and valve seats in Natural-gas injectors. To analyze the factors influencing the joint properties, this paper will first study the welding principles of 303Cu and 440C-Nb dissimilar materials through experiments and numerical simulations. The microstructure and properties of welded joints will be analyzed by the metallographic test, the microhardness test, and EDS. The evolution process of the weld microstructure will be analyzed in combination with the equilibrium phase diagram. A composite heat source model will be established to investigate the distribution of the temperature field and stress field. To improve the mechanical and sealing properties of welded joints, a laser post-heat treatment method is finally proposed in this paper. The method’s effectiveness will also be analyzed by numerical simulation, the mechanical property test, and the helium leak test [21].

## 2. Experiment and Simulation Methods

### 2.1. Sample and Materials

**Figure 1:**
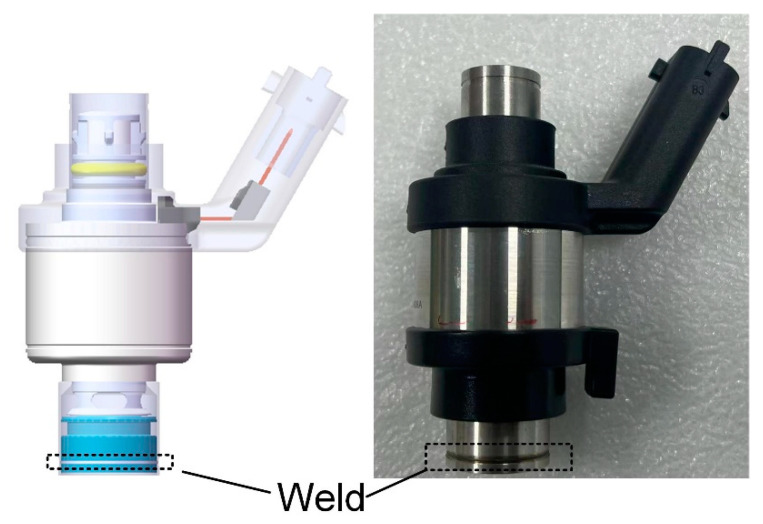
Schematic diagram of test object and welding position.

The valve pipe and seat in the natural-gas injector are combined in a lap joint, and the welding position is shown schematically in Figure 1. The outer diameter of the valve pipe welding area is 18.6 mm, the wall thickness is 0.3 mm, the outer diameter of the valve seat is 18 mm, and the wall thickness is 2 mm. To ensure joint strength and sealing performance under mechanical vibration, a laser beam is used to weld the joint along the outer pipe wall. The chemical composition of the material corresponding to the valve pipe (303Cu) and the valve seat (440C-Nb) is shown in Table 1. The material composition information was derived from reports provided by material suppliers.

### 2.2. Experimental Equipment

**Figure 2:**
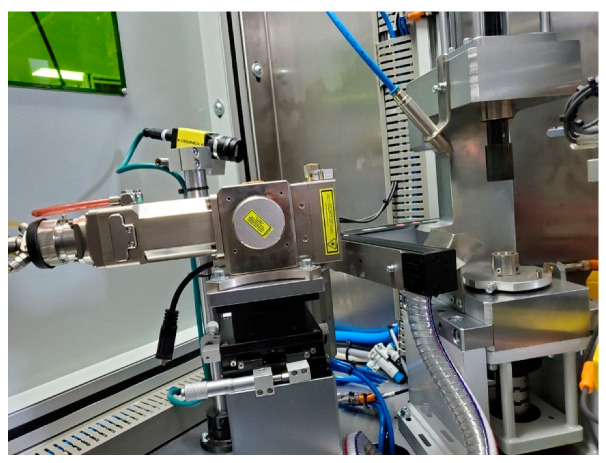
Laser welding system.

**Figure 3:**
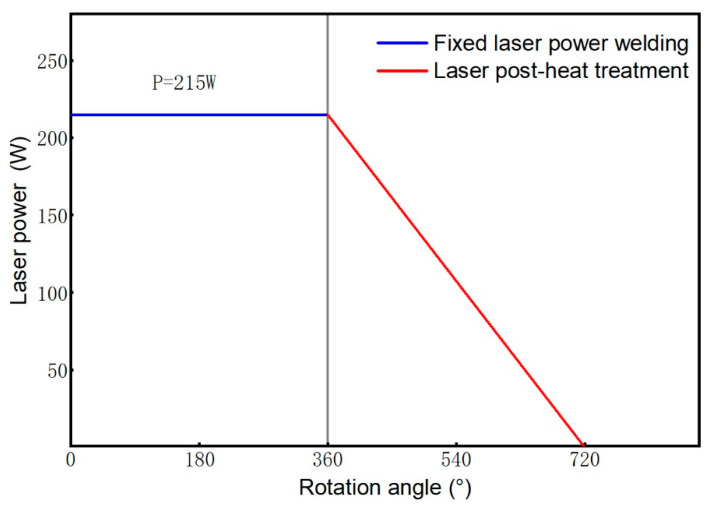
Schematic diagram of the process of laser power reduction.

The welding experiments were carried out by the IPG YLR-500 fiber laser welding system, as shown in Figure 2. The laser focal length is 159 mm, and the fiber diameter is 200 μm. The maximum power is 500 W. The fiber laser and rotary servo workbench are controlled by the programmable logic controller, which can realize fixed power or variable power rotary welding. The pretests determined the adjustment range of process parameters through single-factor experiments: Laser power of 200–300 W, a welding speed of 19.48–29.21 mm/s, and a defocus amount of 0–3 mm. A better combination of process parameters was further determined through orthogonal experiments, as shown in Table 2. This combination of parameters is continuous welding with fixed power in the case of one rotation. The laser post-heat treatment process was also realized by this system. The process was performed immediately after one circle of fixed power welding. The laser power decreased linearly from 215 W to 0 W during one rotation, and the other process parameters remained unchanged, as shown schematically in Figure 3.

**Figure 4:**
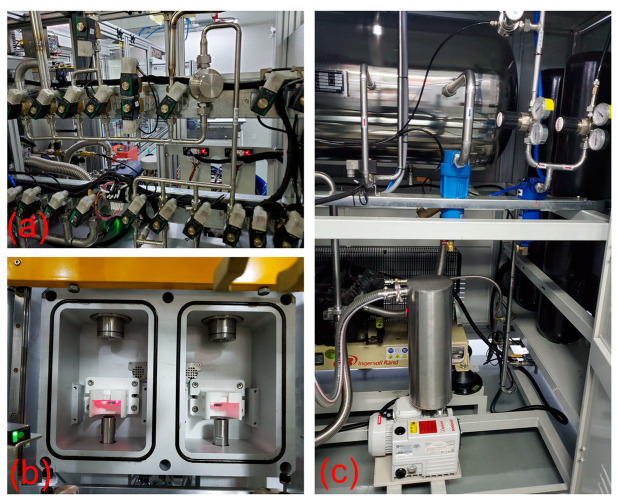
Helium leak test system: (**a**) Helium control valve; (**b**) vacuum test room; (**c**) helium recovery system.

After welding, metallographic samples of the welds were made through a cutting machine (Q-100B). Acrylic resin powder and a curing agent were used to make a cold mosaic sample with a ratio of 1:0.8. A grinding and polishing machine (MP-2B) was used to grind the weld section of the sample, and a 4% nitric acid alcohol solution was used for weld erosion imaging. An optical microscope (SDPTOP ICX4IM, Pooher Electro-Optical Co., Ltd., Shanghai, China) and industrial digital camera (XU3CM0 S0630A, Luxopto Electronics Co., Ltd., Suzhou, China) were used to observe the microstructure of the weld. An electronic scanning microscope and energy spectrometer (Axia ChemiSEM LoVac, Thermo Fisher Scientific Inc., Waltham, MA, USA) were used to analyze the element distribution, and the digital microhardness tester (HVS-1000A, Metallographic Testing Equipment Co., Ltd., Shandong, China) was used to measure the hardness of the weld and the two base metals. The samples after welding and post-heat treatment were put into the electro-mechanical universal testing machine (WANCE-ETM504D, Wance Technologies Ltd., Shenzhen, China) separately for testing. The valve pipe was fixed by specific tooling, the pressing head applied axial pressure to the valve seat, and the mechanical properties of the welded joint were judged by the magnitude of the press-off force. The welding sealing properties test was analyzed by helium leakage test equipment, as shown in Figure 4. The helium leak test system includes a helium pipeline, control valve, vacuum detection chamber, and helium recovery system. When the natural-gas injector was placed in the detection chamber, the device changed to vacuum mode, and then helium gas entered the valve. The natural-gas injector was closed when it was not energized, and the detection device was used to detect the concentration of helium in the vacuum chamber and thus determine the sealing properties of the welded joint.

### 2.3. Numerical Simulation

**Figure 5:**
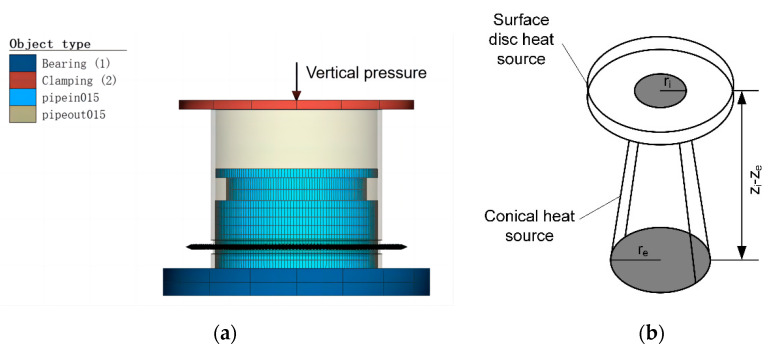
Grid model and laser heat source model: (**a**) Grid model; (**b**) laser heat source model.

In the numerical simulation of laser welding, the surface disk heat source model is usually used to simulate the absorption of laser energy on the metal surface and the simulation of the thermal conduction welding mode. The conical heat source model is used to simulate the keyhole effect and the energy absorption inside the material in the deep penetration welding mode [22,23]. In this study, a composite heat source model of a surface disc heat source and a conical heat source is established by using Simufact Welding software 2020 to simulate the temperature field and stress field in the welding process. The 3D model of the injector and the composite heat source model is shown in Figure 5. The properties of two materials, 303Cu and 440C-Nb, were calculated by JMATPro7.0 software and imported into the model. Hypermesh was used to divide the model into 0.15 mm meshes. The outer valve pipe model was divided into 11,520 meshes, and the inner valve seat model was divided into 21,840 meshes. The mesh refinement process is carried out in the welding area, and the processed mesh size is 0.075 mm, which meets the computational convergence accuracy requirements of the heat source model.

The geometry of the heat source is related to the welding optics, and some calibration is required to obtain more accurate results. The composite heat source model parameters were determined after heat source calibration. The surface disk heat source parameters were a 0.25 mm radius, 0.3 mm depth, and 2.9 Gaussian parameter, while the conical heat source parameters were a 0.2 mm upper radius, 0.55 mm lower radius, 0.5 mm depth, and 1.5 Gaussian parameter. Setting the volume heat flux fraction to 0.9 means that there is surface heat flux in the model. The heat source model can be described by the following equation [24]:

(1) $$Q\left(x,y,z\right)=Q_{0}exp\left(-\frac{x²+y²}{r_{0}^{2}\left(z\right)}\right)$$

Moreover, *r*_0_ (*z*) is defined as follows:

(2) $$r_{0}\left(z\right)=r_{e}+\frac{r_{i}-r_{e}}{z_{i}-z_{e}}\left(z-z_{e}\right)$$

where *Q*_0_ is the maximum volumetric heat flow density; *x*, *y*, and *z* are the heat source coordinates; *r_i_ − r_e_* is the upper and lower cone radius dimensions; *z_i_ − z_e_* is the conical heat source depths.

## 3. Results and Discussion

### 3.1. Principle Analysis of Fixed Power Laser Welding

#### 3.1.1. Verification and Discussion of Numerical Simulation

**Figure 6:**
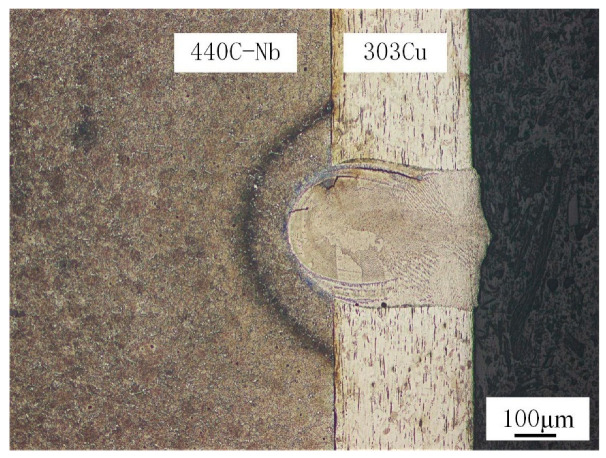
Weld microstructure.

The weld microstructure obtained by the welding test is shown in Figure 6. The width at the top of the weld fusion zone is 0.35 mm, the width at the joint of two layers of material is 0.32 mm, and the penetration is 0.11 mm. The geometric profile of the weld shows that the laser penetrates and melts the outer 303Cu base material, which in turn fuses with the inner 440C-Nb base material to form a cylindrical weld, with an elliptical HAZ observed on the inner 440C-Nb side.

**Figure 7:**
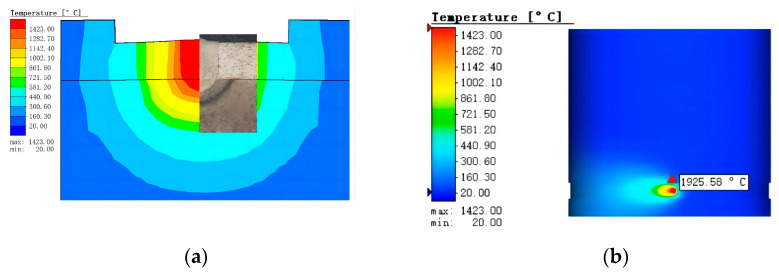
Numerical simulation comparison and temperature field simulation results: (**a**) Comparison of simulation and experimental results; (**b**) temperature field simulation results.

**Figure 8:**
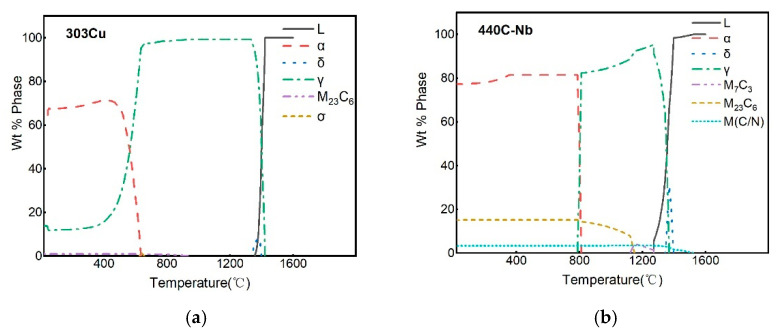
Equilibrium phase diagram of two materials: (**a**) 303Cu; (**b**) 440C-Nb.

According to Kik [25], modeling with a composite heat source model (surface Gaussian heat source and cone heat source) requires modifications to ensure convergence of the model with the actual results, which are very relevant to the temperature and stress distribution in the weld region. The simulated results of the weld region after heat source verification are shown in Figure 7a. The shape and size of the weld section in the simulation and experiment are very similar, which proves the validity of the simulation results. The results of the temperature field simulation are shown in Figure 7b. The maximum temperature of the weld surface can reach 1925 °C, the weld zone can reach 1423 °C, and the HAZ can reach 1142 °C. The equilibrium phase and temperature diagram of the two base materials were calculated by JMATPro7.0 software. The temperature range was set at 20–1600 °C, the step length was 5, and the equilibrium phase diagram was shown in Figure 8.

Compared to Morawiec et al. [26], they analyzed the phase transformation kinetics of materials during the cooling process after laser welding by combining the simulated thermal cycle and JMATPro software. Combined with the temperature field simulation results and the material equilibrium phase diagram obtained, this study also aptly analyzed the microstructure evolution during the cooling process after welding. Under high-energy-density laser irradiation, 303Cu and 440C-Nb base materials are transformed into the liquid phase above the melting point temperature and thus form a molten pool. The molten pool gradually cools and solidifies as the temperature decreases after welding. When the temperature decreases to 1423–1500 °C, the austenite γ phase and a small amount of ferrite δ phase are gradually crystallized from the liquid phase, and some M(C/N) carbides are also crystallized from 440C-Nb. When the temperature decreases to approximately 1350 °C, the δ phase and the remaining liquid phase change into the γ phase, the molten pool is the solid phase, and the γ phase reaches the maximum. This also corresponds to the characteristic that the weld metal takes austenite as the initial phase to solidify in the experiment. As the temperature continues to decrease, small amounts of M_7_C_3_ and some M_23_C_6_ gradually precipitate out of 440C-Nb. The extremely fast laser welding speed leads to a high cooling rate of the melt pool as well as a very short solidification time, which in turn causes the crystallized carbides to fail to reach the dissolution conditions [27]. Therefore, the 440C-Nb material easily precipitates and forms lamellar eutectic carbide during solidification after welding. This further explains the reason for the blocky and island-like distribution of the uneven fusion zone in the 440C-Nb side structure at the bottom of the weld in the experiment.

**Figure 9:**
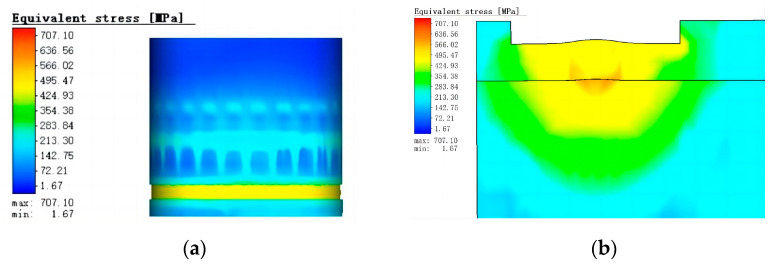
Results of equivalent stress simulation: (**a**) Simulation results of overall equivalent stress; (**b**) simulation results of equivalent stress of weld section.

The simulation results of the stress field in the welding area are shown in Figure 9. The maximum equivalent stress caused by laser welding can reach 707.1 Mpa. Simulation of the stress distribution in the weld section shows that the stress is primarily concentrated in the area where the two materials are joined at the bottom of the weld. The laser welding speed is fast, and the whole solidification rate of the weld metal is high. At the same temperature gradient, due to the different undercooling degrees of the two materials, it is easy to accumulate residual equivalent stress at the material joint in the weld when the cooling rate is high. Therefore, appropriate process methods are needed to reduce the cooling rate after welding.

#### 3.1.2. Microstructure Analysis

**Figure 10:**
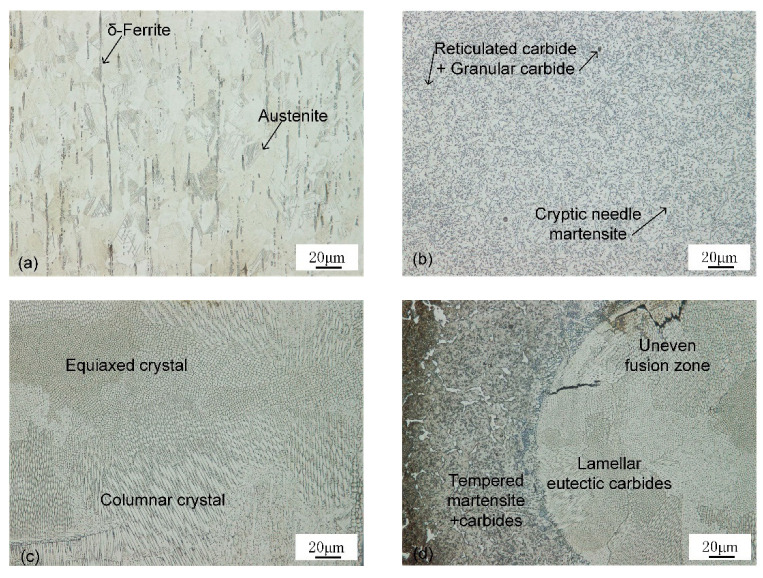
Microstructure analysis of different areas: (**a**) 303Cu BM, (**b**) 440C-Nb BM, (**c**) middle of weld, (**d**) bottom of weld.

Optical microscopic observation reveals the microstructure of the base material, weld seam, and HAZ, as shown in Figure 10. Equiaxed austenite structure and bar δ ferrite formed by chromium segregation were observed in 303Cu base metal (BM). When ferrite exists in austenitic stainless steel, its yield strength is high, but its toughness and ductility may be reduced, and cracks are easily formed during welding [10]. M_23_C_6_ and σ phases may precipitate preferentially on the ferrite phase, whereas the σ phase is a brittle component in stainless steel [28].

Cryptic needle martensite, reticulated massive eutectic carbides, and granular secondary carbides were observed in the 440C-Nb base metal. 440C series steel is a high-carbon, high-chromium martensitic stainless steel prone to the formation of inhomogeneous carbides, resulting in a low degree of alloying of the matrix. After quenching, the martensite is cryptic needle-like, so the matrix is white [29].

As-cast austenite and pitting carbides were observed in the weld structure. The undercooling degree of components at different positions in the weld pool is different, resulting in different crystal forms in the weld [13]. The microstructure at the top of the weld is a primarily columnar crystal with austenite as the matrix. The columnar crystal develops perpendicular to the fusion line to the weld center and meets at the weld center. Due to the final solidification in the weld center, the columnar crystal decreases and the equiaxed crystal increases. Near the bottom of the weld, there is an uneven fusion zone, which is composed of lamellar eutectic carbides distributed in block and island shapes. The microstructure of HAZ of 440C-Nb side material is tempered martensite, retained austenite, and secondary carbide after high-temperature quenching and tempering.

#### 3.1.3. Microhardness Analysis

**Figure 11:**
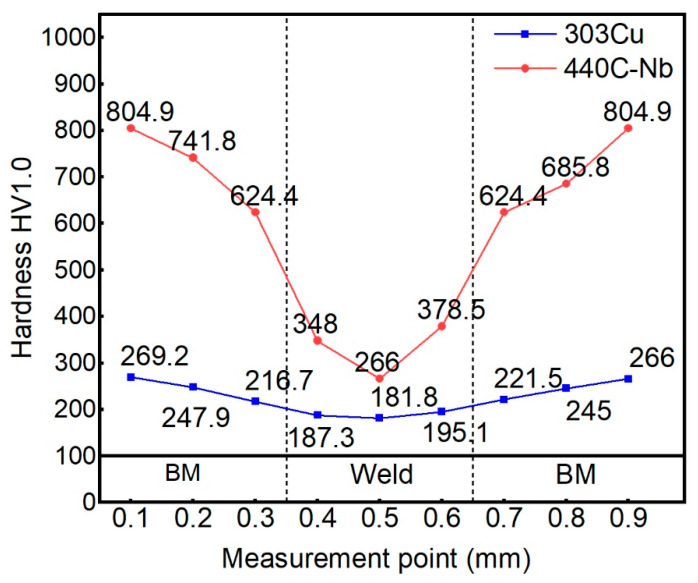
Vickers microhardness analysis.

The hardness of the weld cross-section and all characteristic areas of the BM of the inner and outer layers are tested, and the results are shown in Figure 11. The results of the hardness test showed that the softening phenomenon of weld and HAZ, and the hardness decreases obviously compared with the BM. The difference between the inner and outer base materials can be observed. The hardness of BM at the 303Cu side shall not exceed 270 HV, and the hardness gradually decreases to the minimum value of 181.8 HV as it approaches the middle of the weld. The maximum hardness of the BM at the 440C-Nb side is 804.9 HV, which also decreases significantly with the hardness near the middle of the weld. The hardness of the HAZ decreased to 624.4 HV, and the bottom of the weld was 266 HV, with strengthening occurring relative to the top of the weld. The reduction and deletion of martensite in the HAZ and weld are the main reasons for the hardness reduction, respectively.

#### 3.1.4. EDS Analysis

**Figure 12:**
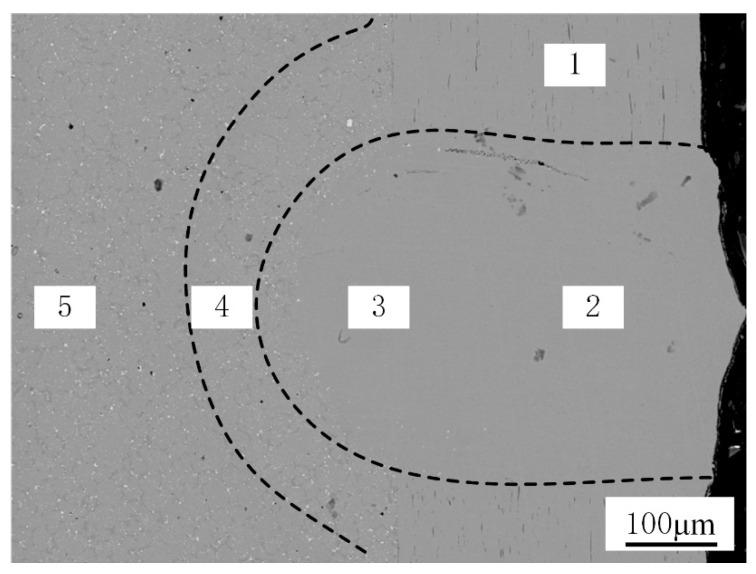
Schematic diagram of EDS measurement area: (1) 303Cu BM, (2) top of weld, (3) Bottom of weld, (4) Heat-affected zone, (5) 440C-Nb BM.

**Figure 13:**
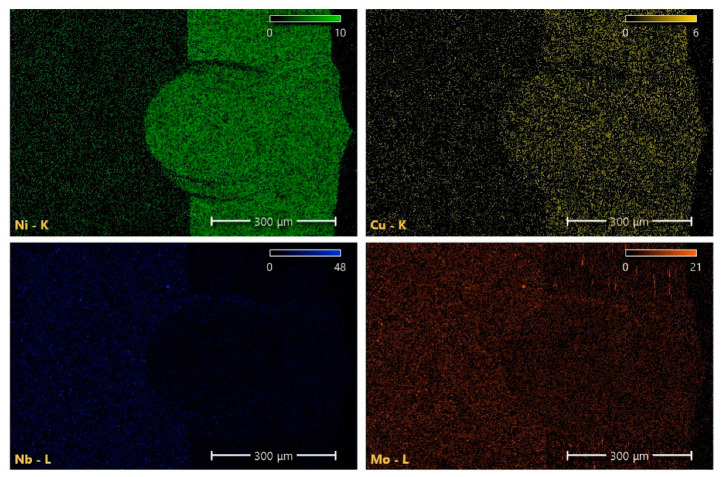
Element map of the weld area.

As shown in Figure 12, the chemical composition of the micro-region was measured, and the distribution of the main alloy composition along the cross-section of the weld bead and the two BMs were analyzed. The proportion of measured alloy elements is shown in Table 3. Because the test area of the BM is closer to the boundary of the weld, the micro-area composition will change under the temperature-enhanced diffusion phenomenon. The element mapping in the weld region is similar to the composition of the 303Cu BM, and the composition near the fusion line is similar to that of the 440C-Nb BM, as shown in Figure 13. It is observed that the alloying elements Cu and Ni diffuse from austenitic stainless steel to the weld. As the austenitic forming elements, Cu and Ni are primarily used to promote the formation of the austenite phase, but Ni is not the forming element of strengthening carbides, nor does it promote the formation of intermetallic compounds [30]. Therefore, the main structure of the upper part of the weld is columnar dendrite and equiaxed crystal based on austenite.

The decrease in Ni and Cu and the increase in Nb and Mo could be observed as the weld test area approached the 440C-Nb side. It is primarily due to the enhanced diffusion mechanism caused by the composition gradient, but it also shows that carbides and intermetallic compounds easily form near the fusion line. Nb and Mo can prevent intergranular corrosion but can also promote the formation of MC carbides [9]. This type of carbide is not easy to dissolve in the welding process, resulting in a blocky or island-like uneven fusion zone at the bottom of the weld.

Combined with the analysis of microstructure and EDS, it is found that the weld metal takes the austenite as the initial phase to solidify. Because of the weak diffusivity of alloying elements and impurities in austenite at high temperatures, it is easy to cause microsegregation [18,31]. A large weld produced by high heat input or a teardrop-shaped weld formed by excessive welding speed can easily contribute to a solidification crack [32]. Therefore, the welding process with high restraint on the solidified weld metal will increase its solidification crack sensitivity. It is necessary to employ an appropriate post-heat treatment process to reduce the cooling rate of the weld and improve the microstructure and properties of the welded joint.

### 3.2. Comparative Analysis of Laser Post-Heat Treatment

#### 3.2.1. Comparison of Stress and Temperature Simulation

**Figure 14:**
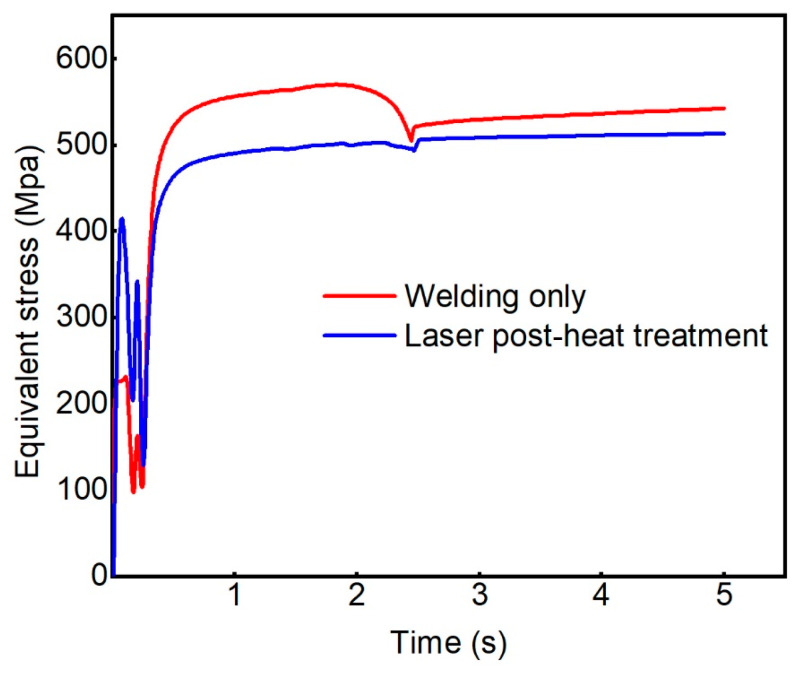
Residual equivalent stress history diagram of weld zone in different processes.

**Figure 15:**
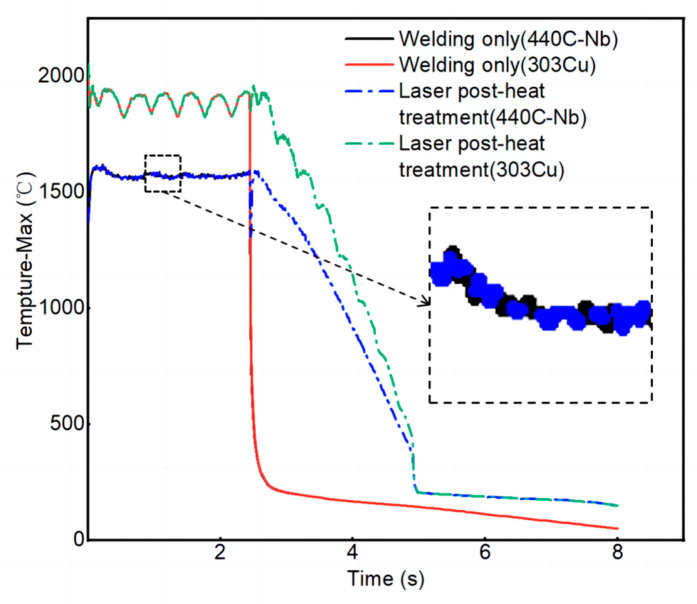
Maximum temperature history chart of different processes.

As the equivalent stresses are primarily concentrated in the joint between two materials in the weld, the stresses in the material joint in the weld are monitored. The stress data of only one circle of laser welding and laser post-heat treatment are analyzed, and the comparison results are shown in Figure 14. After laser post-heat treatment, the equivalent stress at the joint of the two materials in the weld can be reduced to a certain extent. The temperature history of the inner and outer materials during welding and cooling was simulated, as shown in Figure 15. The temperature of the outer layer is higher than that of the inner layer because the heat is transferred to the inner layer after laser irradiation. After laser post-heat treatment, the cooling rate after welding is obviously reduced. At the same temperature gradient, the cooling mode changes from fast to slow and then reduces the stress in the weld. Kik et al. [33] proved that the residual stress is related to the temperature gradient and cooling rate. The metal material obtains the time to release stress during slow cooling, thus the residual stress is reduced. This also provided an explanation for the reduction of residual stress in welded joints after laser post-heat treatment in this test.

#### 3.2.2. Comparison of Mechanical Properties

**Figure 16:**
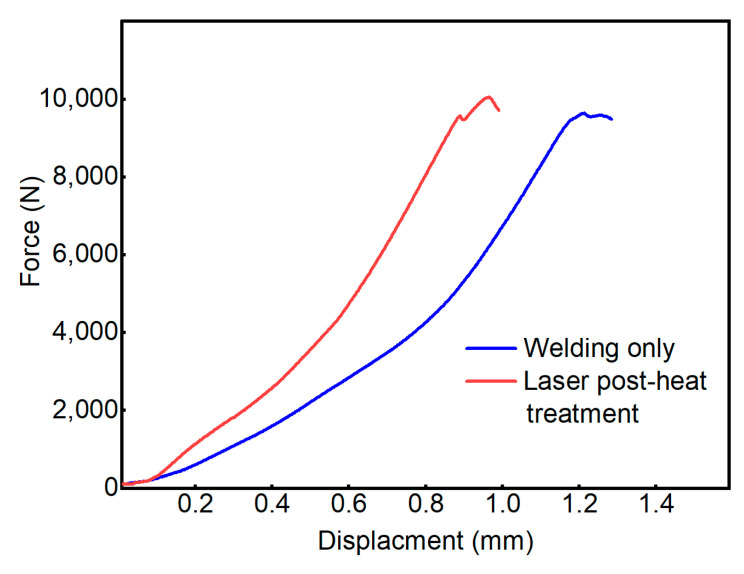
Force–displacement curves.

The mechanical properties of the welded joints were tested, and the force–displacement curves obtained are shown in Figure 16. The maximum press-off force obtained after only one circle of welding at a fixed laser power was 9640 N. The mechanical strength of the weld obtained met the joint strength requirements. After laser post-heat treatment, the maximum mechanical strength of the weld reaches 10,046 N. After one circle of normal welding, we added one circle of laser post-heat treatment with gradually decreasing power, which slightly increases the heat input of the whole welded joint. Compared with the case of only one circle of welding, laser post-heat treatment can slightly improve the mechanical properties of the weld under the condition of meeting the joint strength of the weld.

#### 3.2.3. Comparison of Sealing Properties

**Figure 17:**
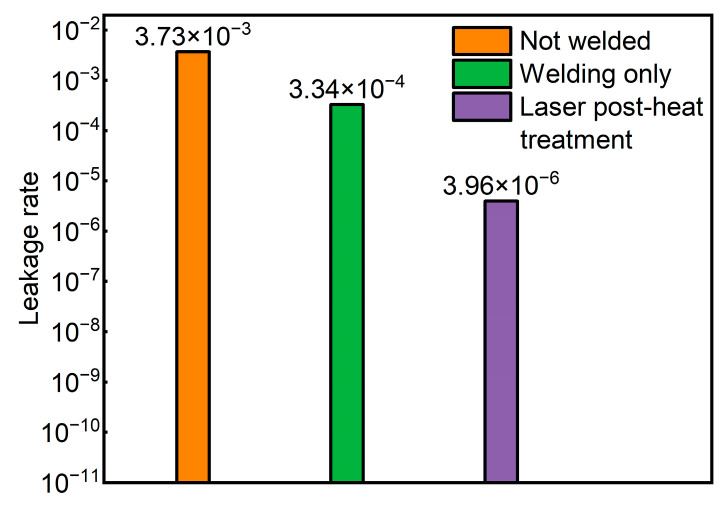
Helium leak test.

The test results of sealing performance through the helium leakage test are shown in Figure 17. The leak test shall be conducted before welding the sample to ensure that there is no leakage caused by other factors. Since there is no sealing weld between the two materials before welding, the leakage rate before welding exceeds the leakage threshold. Laser welding melted the outer valve pipe and the inner valve seat to form a circle of the weld. In the case of only one circle of welding with fixed power, due to the rapid cooling rate of the weld structure, it is easy to produce cracks due to uneven fusion areas or residual stress. The welded joint obtained in this case has a certain sealing performance, but there is still some gap between the good sealing performance. Analysis of the results of the comparative test shows that the sealing performance of the welded joint is improved to a greater extent after the laser post-heat treatment with slow power reduction compared to the case of only one circle of laser welding. In the case of a weld around a circle, where the weld start and end points overlap, a laser post-heat treatment with a slow power reduction reduces the weld heat buildup. Combined with the stress field simulation results and welding mechanism analysis, the post-heat treatment reduces the cooling rate in the weld area, decreases the equivalent stress at the material joint within the weld, reduces the tendency of cracking at the welded joint, and thus improves the sealing performance of the welded joint.

## 4. Summary

In the fixed power laser welding of 303Cu and 440C-Nb dissimilar stainless-steel metals, cylindrical welded joints were obtained. A composite heat source model consisting of a surface disk heat source and a conical body heat source was established based on Simufact Welding, and simulation results were obtained very closely to the experiments. Combined with the equilibrium phase diagram and temperature field simulation results, the microstructure evolution of the material during laser welding was analyzed. The weld metal was solidified primarily with austenite as the initial precipitation phase. The stress field simulation shows that residual equivalent forces tend to concentrate at the material joints within the weld.

The microstructure analysis shows that due to the different cooling rates and undercooling degrees, the top of the weld is primarily columnar crystal, the center of the weld is equiaxed crystal, and the bottom of the weld appears to be an uneven fusion zone. The hardness of the weld and HAZ decreases greatly relative to the BMs, and the hardness decreases significantly as it approaches the center of the weld. The hardness of 440C-Nb is greater than 303Cu, thus causing the top of the weld hardness to be less than the bottom. The migration of Nb and Mo elements in the 440C-Nb to the weld resulted in the formation of lamellar eutectic carbides with blocky and island distributions at the bottom of the weld.

The analysis of the welding principle shows that a cooling rate that is too fast after welding leads to the existence of residual stress and uneven fusion at the bottom of the weld, which are the main factors affecting the properties of the welded joint. The numerical simulation shows that the laser post-heat treatment process can slow down the cooling rate and reduce the residual equivalent stress in the welded joint. The comparative analysis of mechanical properties and leakage test shows that the laser post-heat treatment process can obtain good mechanical properties of the weld and effectively improve the sealing performance of the weld.

## Figures and Tables

**Table 1:** Chemical composition of materials.

Weight (%)										
Materials	C	Si	Mn	P	S	Cr	Ni	Mo	Cu	Nb
303Cu	0.05	0.09	2.37	0.026	0.272	17.41	8.54	-	2.03	-
440C-Nb	1.20	0.60	0.80	-	-	17.00	0.15	0.50	-	3.00

**Table 2:** Laser welding process parameters.

Laser Power (W)	Welding Speed v/(mm/s)	Defocus Amount (Δf/mm)	Rotation Angle (°)
215	23.88	+1.5	360

**Table 3:** Comparison of alloy element composition in each area.

Weight (%)					
Elements	Area 1	Area 2	Area 3	Area 4	Area 5
Ni	7.7	7.2	7.4	2.3	0.3
Cu	2.0	1.7	1.5	0.0	0.0
Nb	0.0	0.0	0.0	2.6	3.8
Mo	0.0	0.0	0.0	0.6	0.4

## Data Availability

Not applicable.
